# Supplementary material for: Production of Astaxanthin by Animal Cells via Introduction of an Entire Astaxanthin Biosynthetic Pathway
Source: Bioengineering (Basel). 2023 Sep 11;10(9):1073. doi: 10.3390/bioengineering10091073 (PMC10525450; doi:10.3390/bioengineering10091073)
Supplement: Supplementary file 1 [file bioengineering-10-01073-s001.zip › Supplementary-Figure S1. RP- HPLC chromatogram of Ast and Can standards and Control sample.pdf]

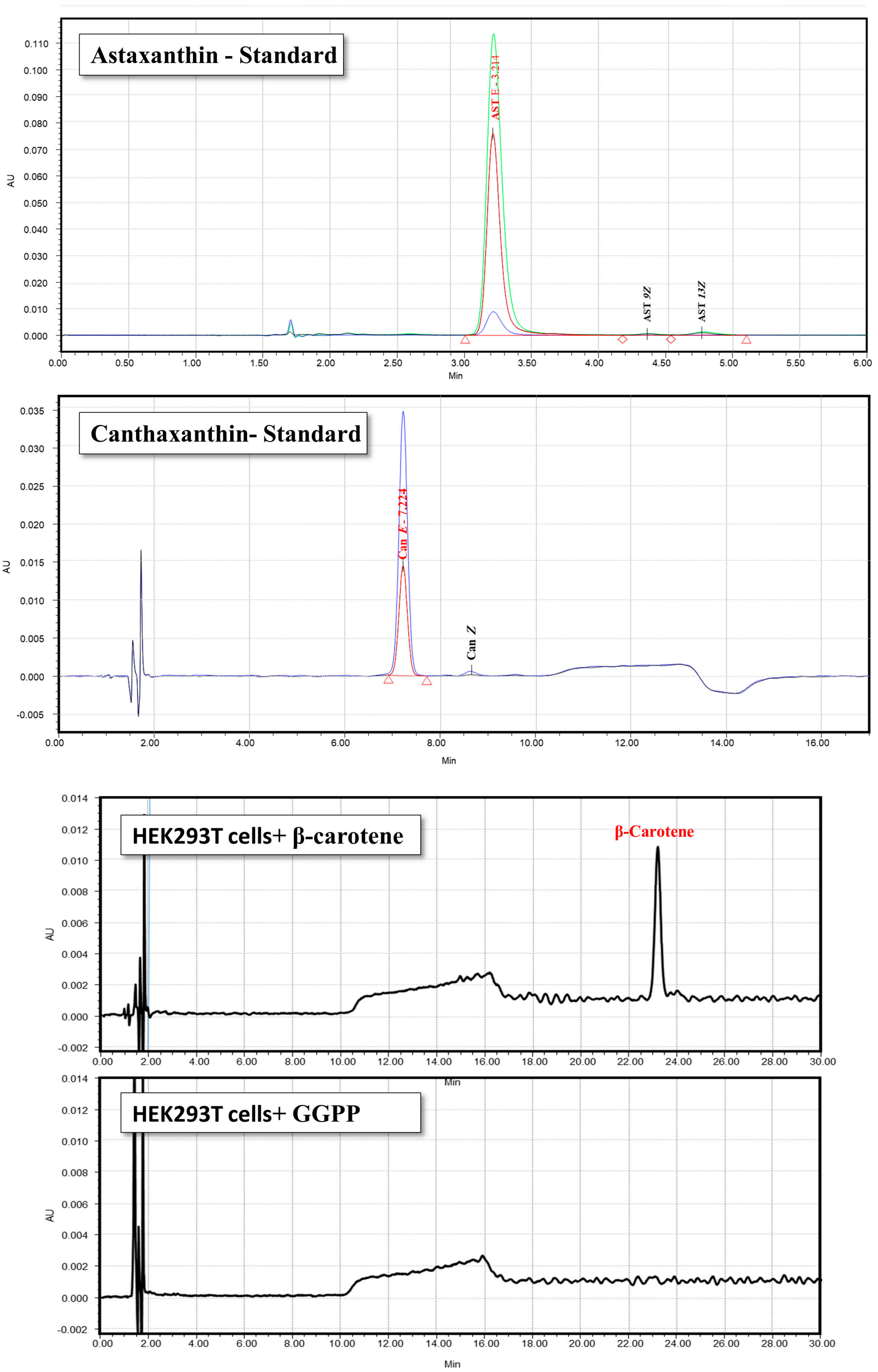

Fig S1. RP- HPLC chromatogram of Astaxanthin (Ast) and Canthaxanthin (Can); *E*, *trans* geometric isomer; *Z*, *cis* geometric isomer; Control sample for HEK293T cells supplied with β-carotene, and GGPP.
