# Supplementary material for: Production of Astaxanthin by Animal Cells via Introduction of an Entire Astaxanthin Biosynthetic Pathway
Source: Bioengineering (Basel). 2023 Sep 11;10(9):1073. doi: 10.3390/bioengineering10091073 (PMC10525450; doi:10.3390/bioengineering10091073)
Supplement: Supplementary file 1 [file bioengineering-10-01073-s001.zip › Supplementary-Figure S2. Animal endogenous mevalonate pathway.pdf]

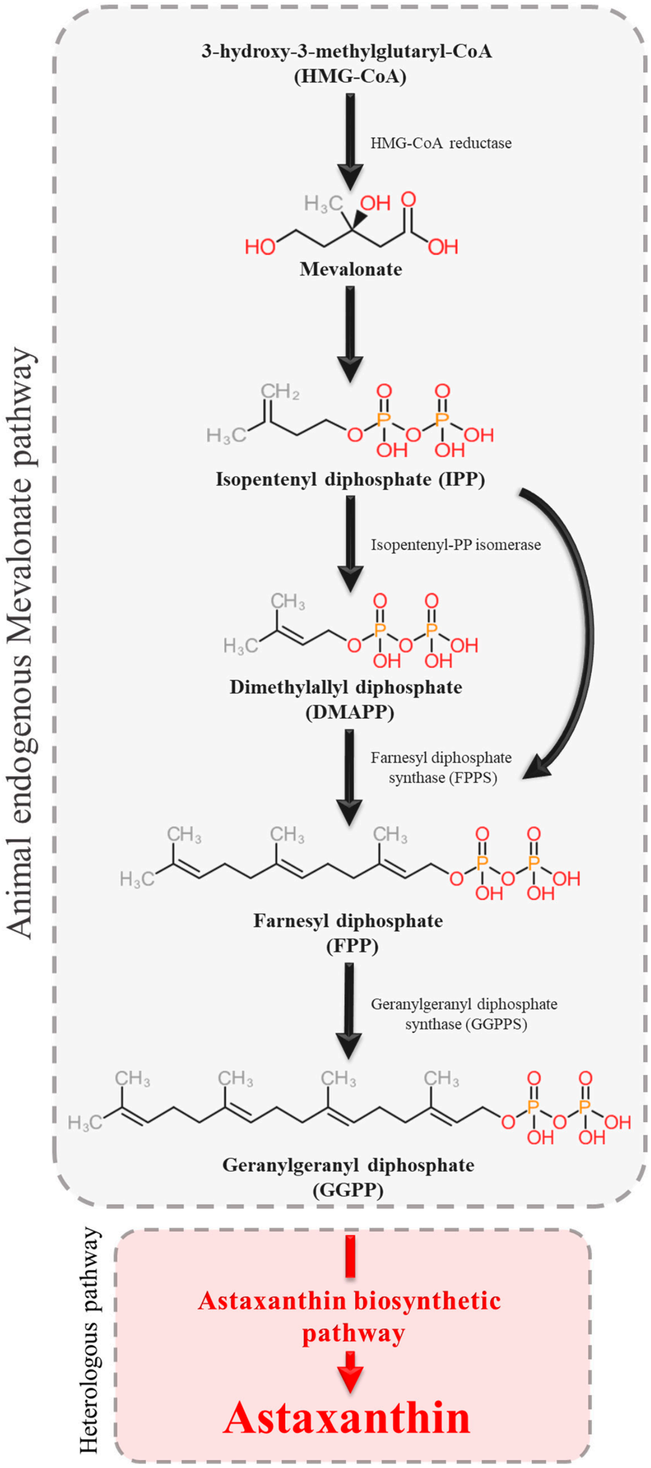

Fig S2. Animal endogenous mevalonate pathway and GGPP flux redirection toward astaxanthin accumulation in HEK293T cells.
