# Supplementary material for: Production of Astaxanthin by Animal Cells via Introduction of an Entire Astaxanthin Biosynthetic Pathway
Source: Bioengineering (Basel). 2023 Sep 11;10(9):1073. doi: 10.3390/bioengineering10091073 (PMC10525450; doi:10.3390/bioengineering10091073)
Supplement: Supplementary file 1 [file bioengineering-10-01073-s001.zip › Supplementary-Figure S3. Predictive 3D of protein modeling, using Phyre2 web portal.pdf]

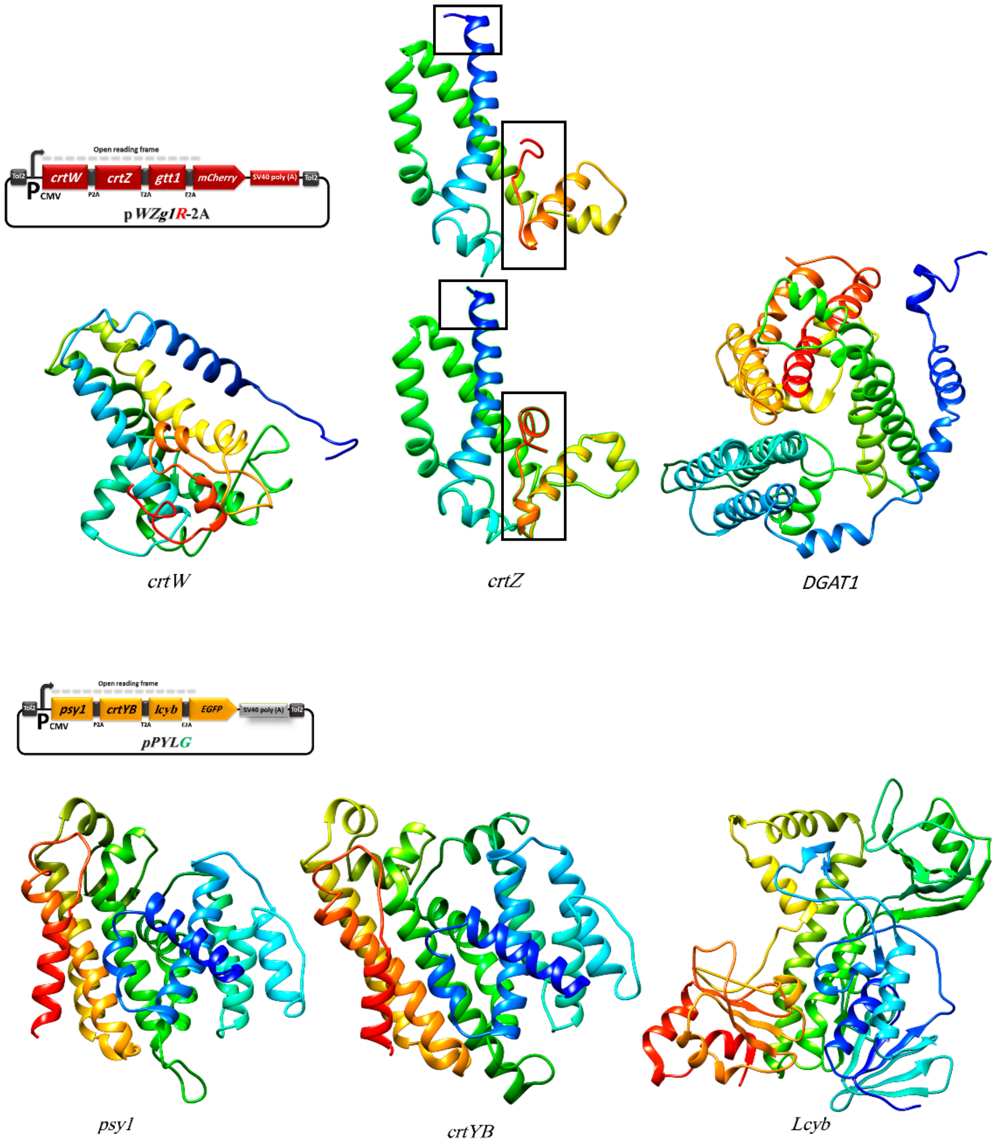

Fig S3. Predictive 3D model of CrtW, CrtZ, and DGAT1 protein modeling translated from p*WZg1*-2A plasmid and psy1, crtYB, and lcyb protein modeling translated from pPYLG plasmid. Prediction and analysis of enzymes were obtained using the Phyre2 web portal (Kelley et al., 2015). The multicistronic expression vector has resulted in a slight change in crtZ conformation near the P2A region. The first helix is very close to the N-terminal (12 amino acid) and this helix is predicted to be involved in the active site, and thus inhibit the enzyme from function correctly.
