# Supplementary material for: Production of Astaxanthin by Animal Cells via Introduction of an Entire Astaxanthin Biosynthetic Pathway
Source: Bioengineering (Basel). 2023 Sep 11;10(9):1073. doi: 10.3390/bioengineering10091073 (PMC10525450; doi:10.3390/bioengineering10091073)
Supplement: Supplementary file 1 [file bioengineering-10-01073-s001.zip › Supplementary-List of synthetic genes and plasmids involved in the study.pdf]

List of synthetic genes and plasmids involved in the study:

1. ***bkt3*** **gene (GenBank: AY603347) from** ***Haematococcus pluvialis*****;**

**Primers**	
**Forward**	CCATCGATATGCACGTGGCTAGCGCTCT
**Reverse**	CCCAAGCTTAGCCAGAGCAGGCACCAGT

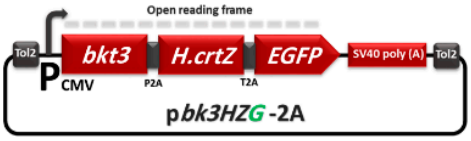

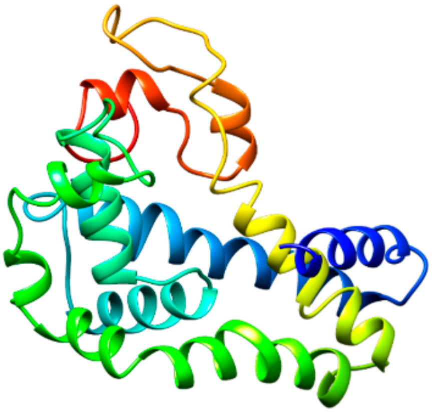

2. H-crtZ gene (GenBank: AY187011) from *Haematococcus pluvialis*

Primers	
Forward	CCCAAGCTTGGATCCGGAGCCACGAACTTCTCTCTGTTAAAGCAAGCAGGAG ACGTGGAAGAAAACCCCGGTCCTCTCAGCAAGCTGCAGAGCAT
Reverse	CGGAATTCTCTTTTGCTCCAGTCCAGTT

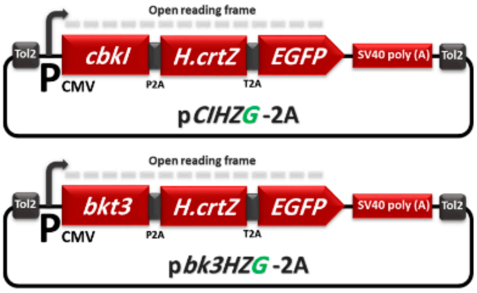

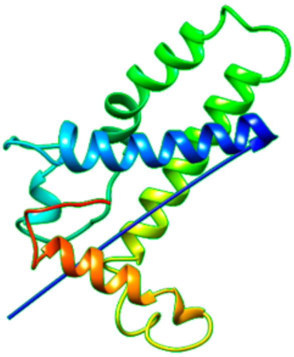

3. CBK1 gene (RefSeq: XM_001698647) from *Chlamydomonas reinhardtii*

Primers	
Forward	CCATCGATATGGGACCTGGAATCCAGCCGA
Reverse	CCCAAGCTTAGCCATAACCCCCACAGGA

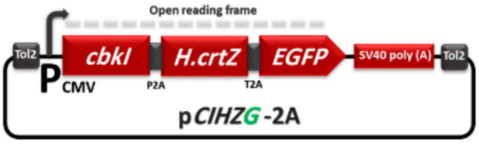

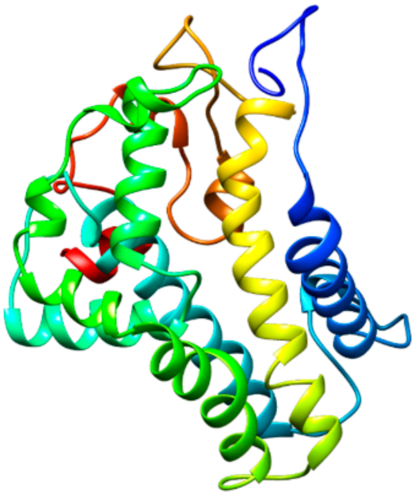

4. DGTT1 gene (GenBank: MN561785.1) from *Haematococcus lacustris*

**Primers**	
**Forward**	GGACTAGTGGATCCGGAGAGGGCAGGGGAAGTCTTCTAACATGCGGGGACGTGGA GGAAAATCCCGGCCCCAGAATCATGCCCTGGCCTTT
**Reverse**	CCGCTCGAGGATCACGATTCCAGAGCTTGT

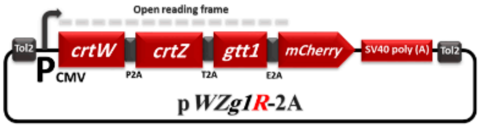

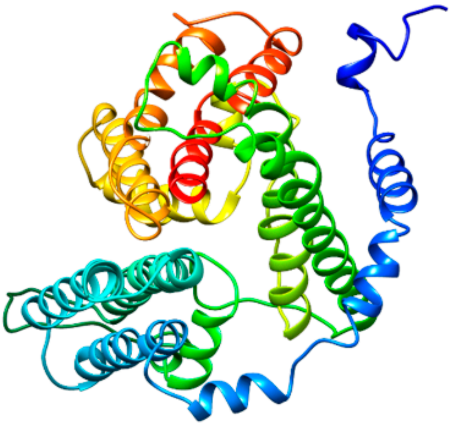

5. psy1 gene (GenBank: FJ971252.1) from *Zea mays subsp*

**Primers**	
**Forward**	CCATCGATatggcaatcatcctggtgaga
**Reverse**	CCCAAGCTTTGTCTGTCCGTTTCTCAGGCT

**Primers**	CCCAAGCTTGGATCCGGAGCCACGAACTTCTCTCTGTTAAAGCAAGCAGGAGACGTG GAAGAAAACCCCGGTCCTATGGCAATCATCCTGGTGAGA
**Forward**	CGGAATTCTCATGTCTGTCCGTTTCTCAG

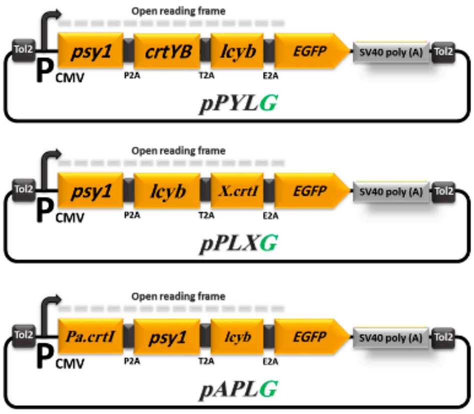

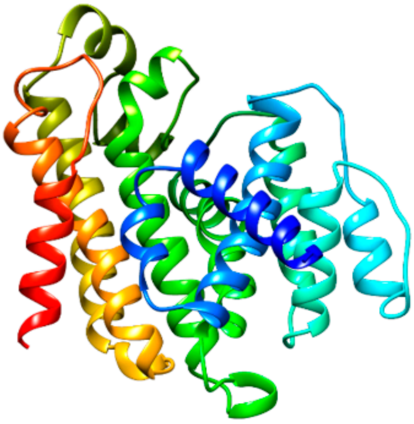

6. lcyb gene (GenBank: KX424526.1) from *Haematococcus lacustris*

Primers	
Forward	CGGAATTCGAGGGCAGGGGAAGTCTTCTAACATGCGGGGACGTGGAGGAAAATCCC GGCCCCATGCTGAGCCCTCTGCAGAGACT
Reverse	CGGGATCCTCACTTGATCATAGCGGGTGGG

Forward	CCCAAGCTTGGATCCGGAGCCACGAACTTCTCTCTGTTAAAGCAAGCAGGAGACGTG GAAGAAAACCCCGGTCCTATGCTGAGCCCTCTGCAGAGA
Reverse	CGGAATTCTCACTTGATCATAGCGGGTGG

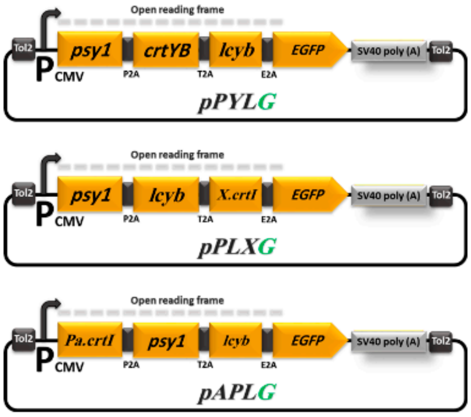

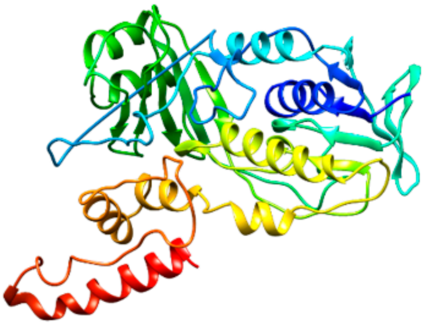

7. Pa-crtI gene (GenBank: D90087.2) from *Pantoea ananatis*

**Primers**	
**Forward**	ccatcgatatgaagcccacaaccgtgatc
**Reverse**	CCCAAGCTTTCAGATCAGGTCTTCCAGCAT

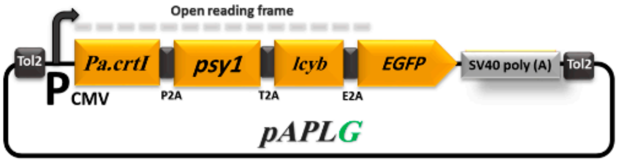

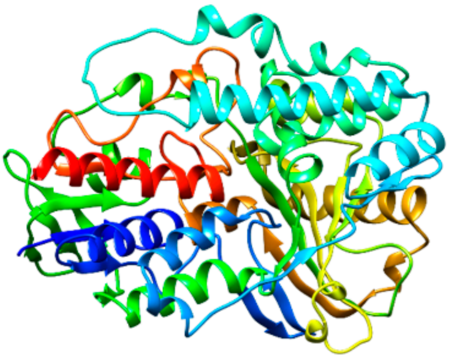

8. Xd-crtI gene (GenBank: KR779666.1) from *Xanthophyllomyces dendrorhous*,

**Primers**	
**Forward**	CGGAATTCGAGGGCAGGGGAAGTCTTCTAACATGCGGGGACGTGGAGG AAAATCCCGGCCCCATGGGAAAGGAGCAGGACCAGGA
**Reverse**	CGGGATCCTCAGAAAGCCAGCACTCCCAC

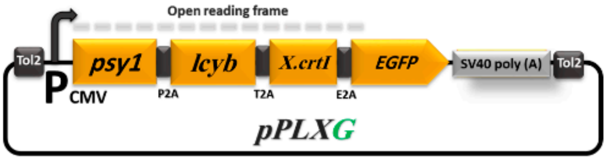

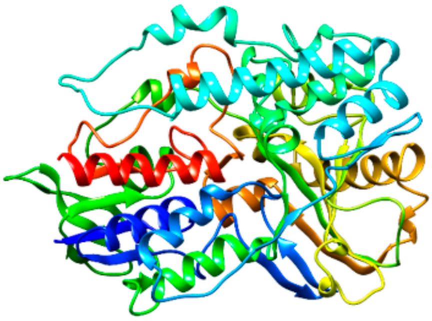

9. crtYB gene (GenBank: AY177204.1) from *Xanthophyllomyces dendrorhous*

Primers	
Forward	CCCAAGCTTggatccggagccacgaacttctctctgttaaagcaagcaggagacgtggaagaaaaccccggt cctacagccctggcctactacca
Reverse	CGGAATTCCTGTCCCTCCCATCCACTCA

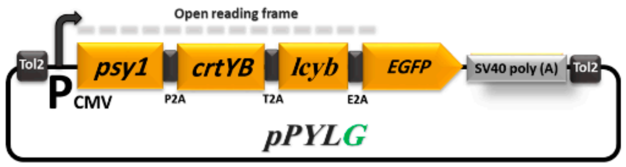

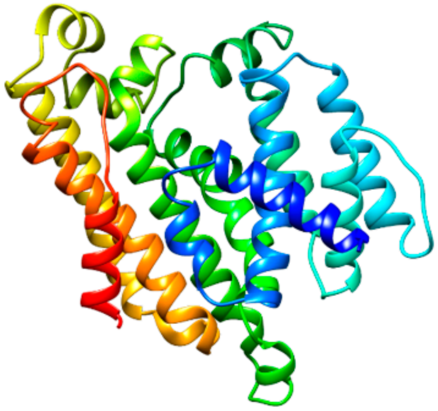

10. Self-cleaving 2A peptides coding sequence:

- **P2A peptides**

GGATCCGGAGCCACGAACTTCTCTCTGTTAAAGCAAGCAGGAGACGTGGAAGAAAACC
CCGGTCCT

GSGATNFSLLKQAGDVEENPGP

- **T2A peptides**

GGATCCGGAGAGGGCAGGGGAAGTCTTCTAACATGCGGGGACGTGGAGGAAAATCCCG
GCCCC

GSGEGRGSLLTCGDVEENPGP

- **E2A peptides**

GGATCCGGACAGTGTACTAATTATGCTCTCTTGAAATTGGCTGGAGATGTTGAGAGCAAC
CCAGGTCCC

GSGQCTNYALLKLAGDVESNPGP
