# Supplementary material for: Production of Astaxanthin by Animal Cells via Introduction of an Entire Astaxanthin Biosynthetic Pathway
Source: Bioengineering (Basel). 2023 Sep 11;10(9):1073. doi: 10.3390/bioengineering10091073 (PMC10525450; doi:10.3390/bioengineering10091073)
Supplement: Supplementary file 1 [file bioengineering-10-01073-s001.zip › Supplementary-RP- HPLC chromatogram of ketocarotenoids for HEK293T cell-extracts.pdf]

➢ **RP-HPLC chromatogram of ketocarotenoids for HEK293T cell extracts**

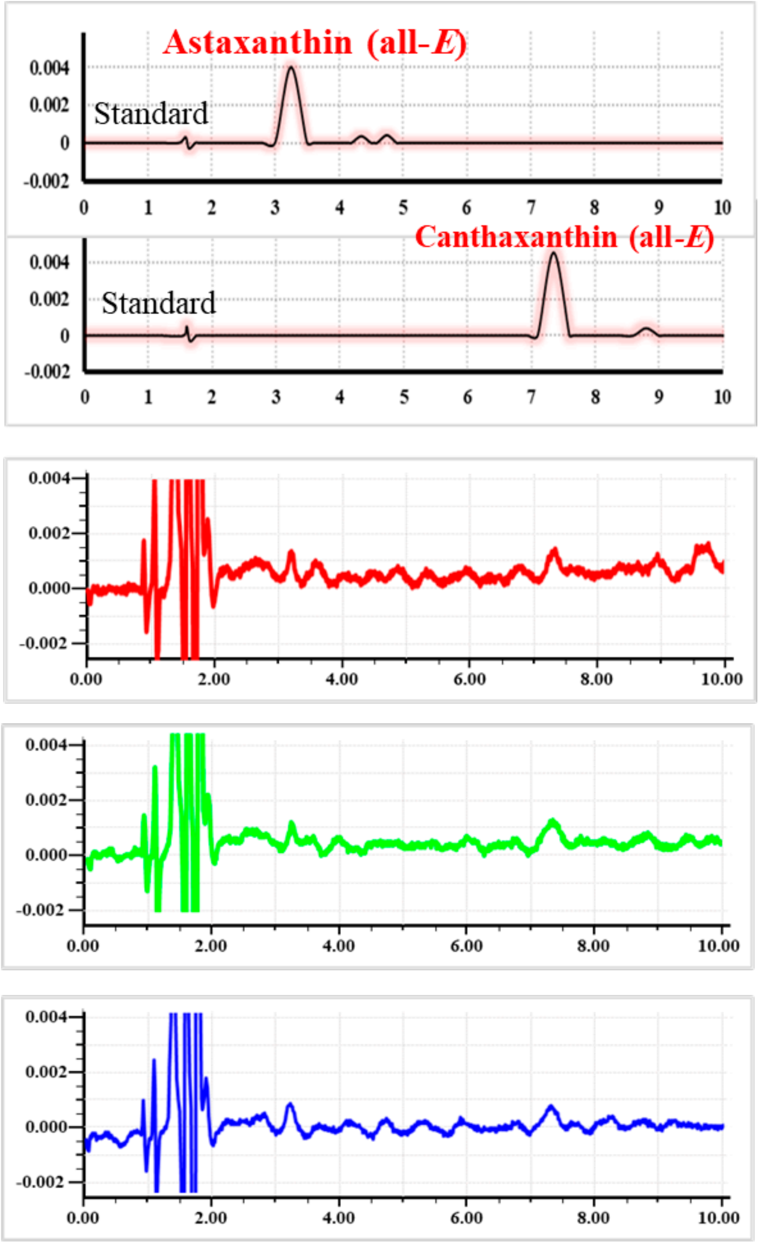

1- pWZG-2A+beta-carotene

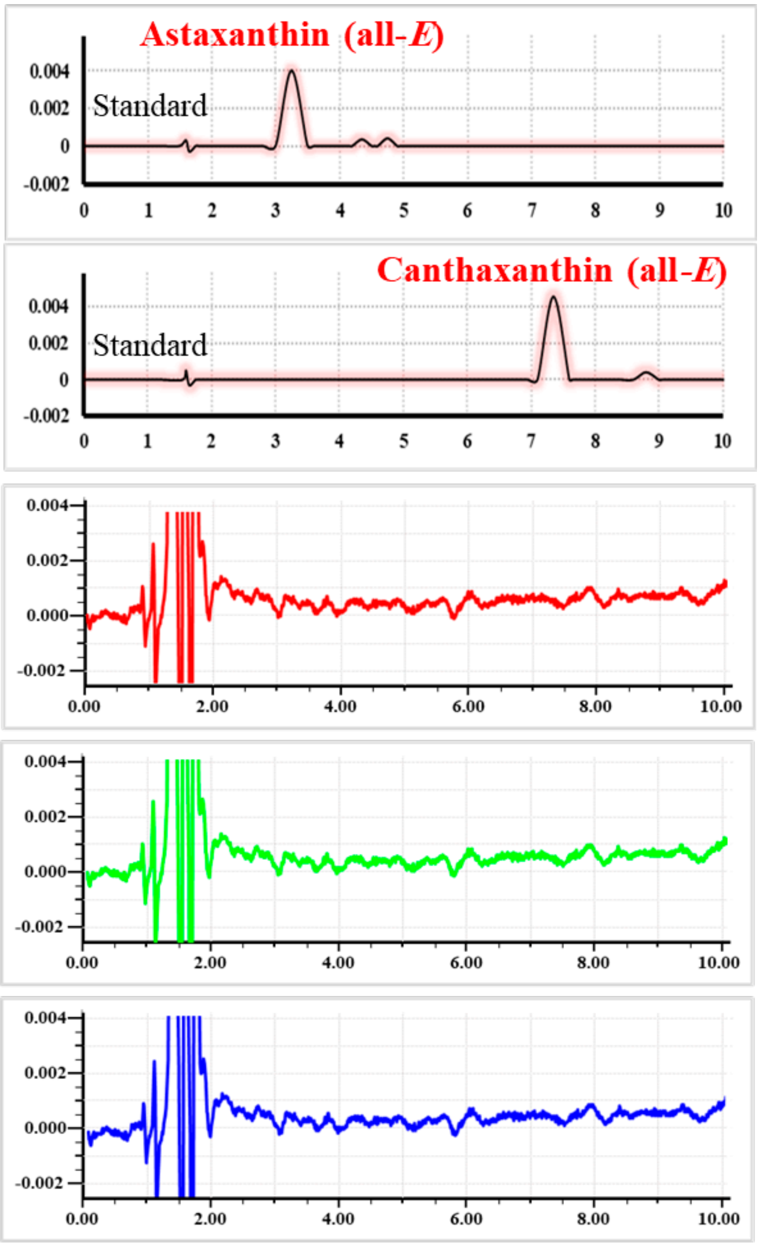

2- pbK3HZ-2A+beta-carotene

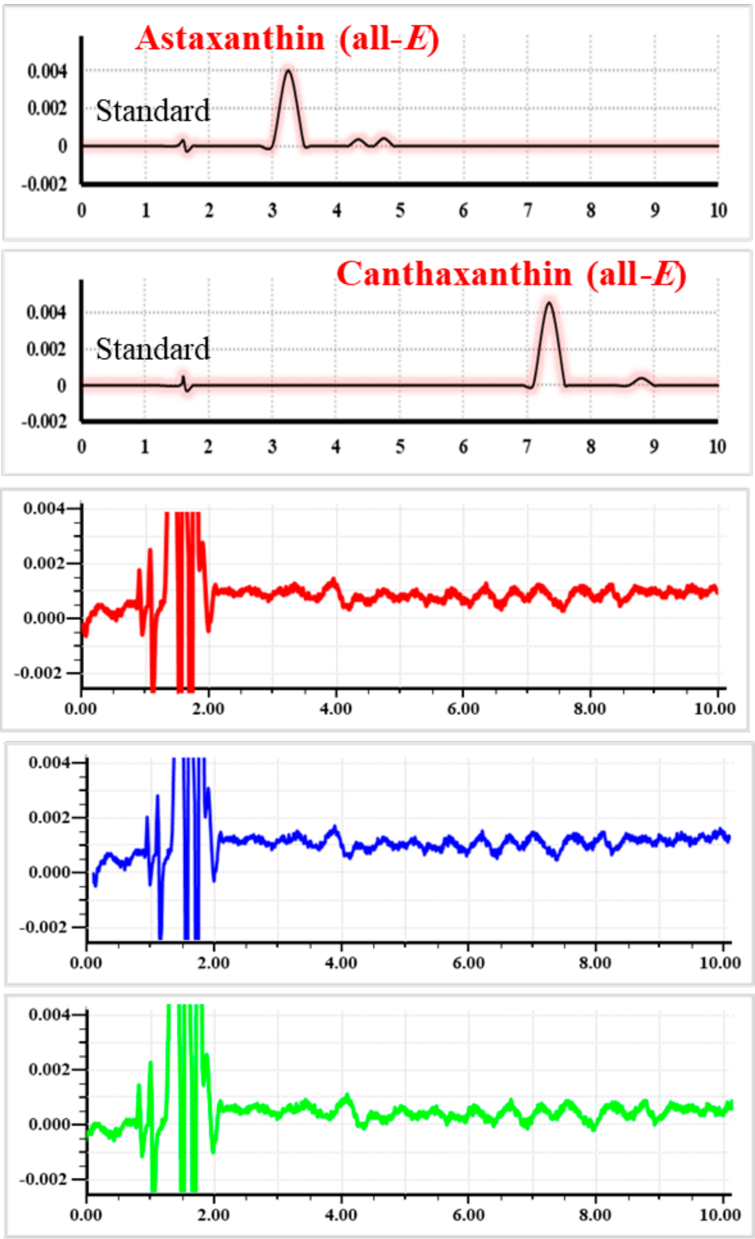

3- pC1HZG-2A+beta-carotene

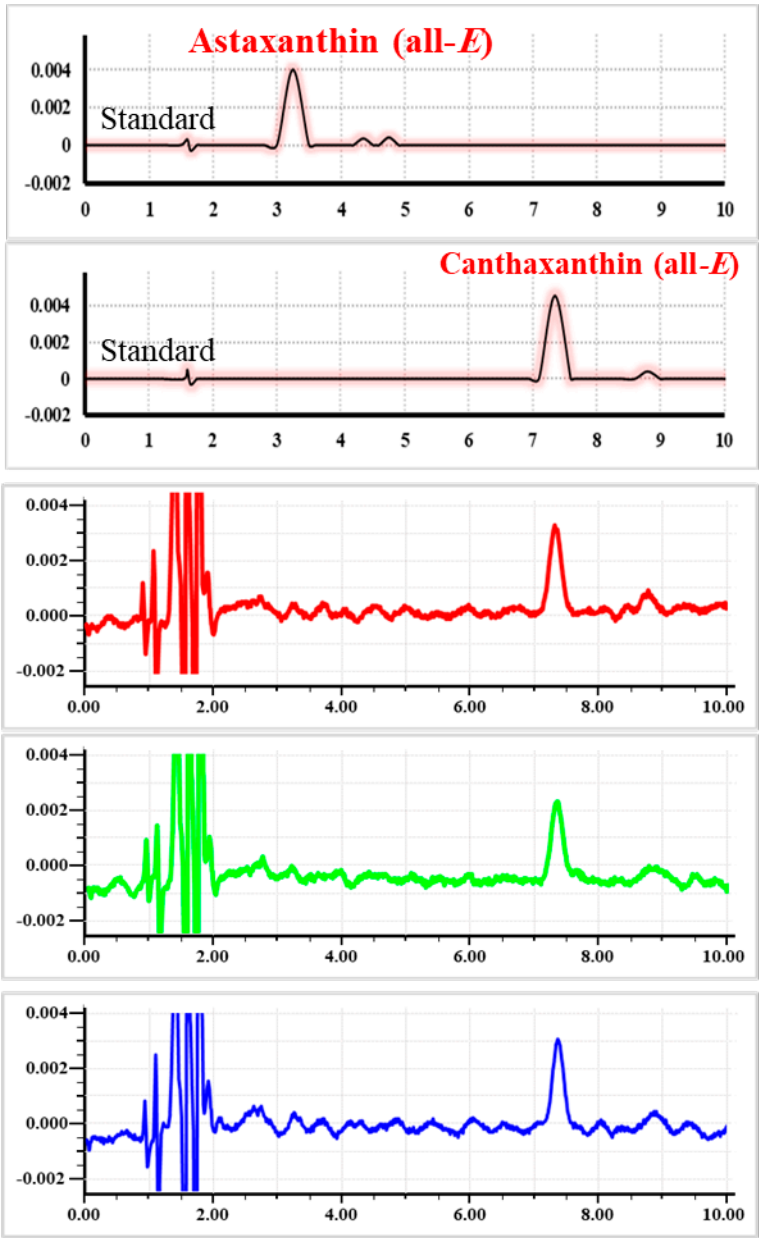

4- pWZg1R-2A+beta-carotene

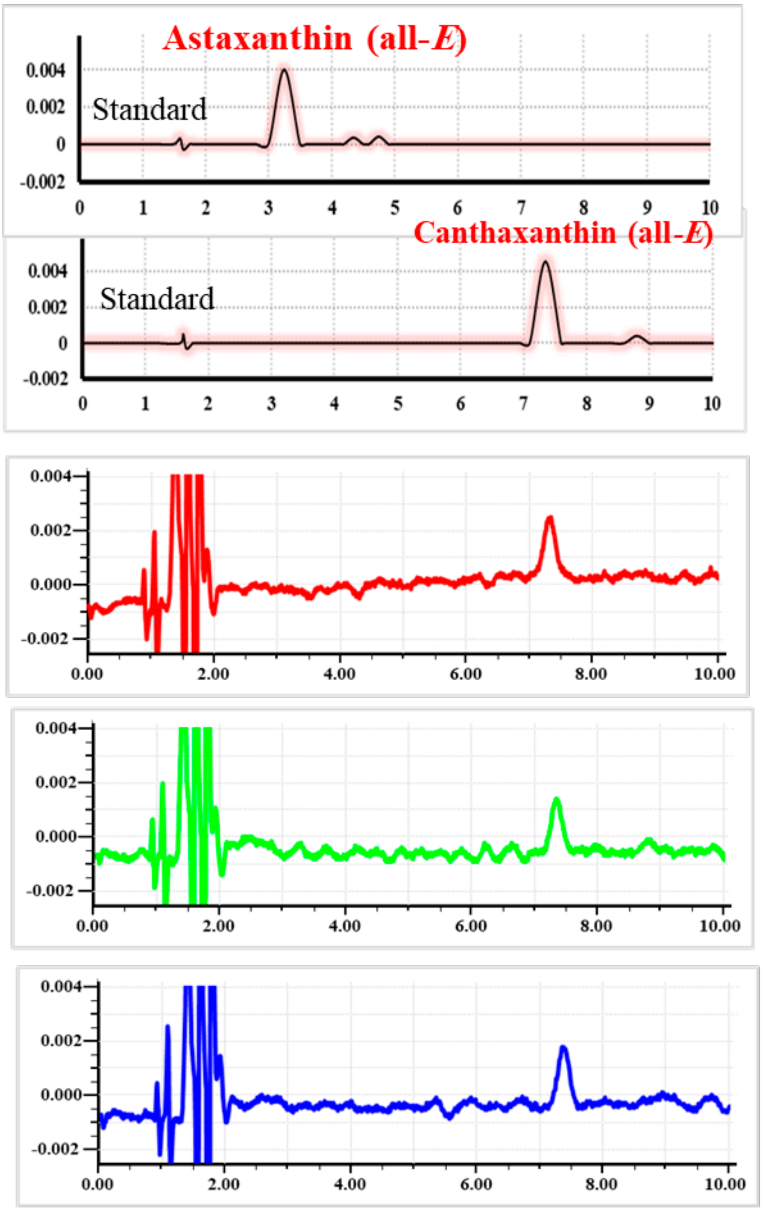

5- pWZg2R-2A+beta-carotene

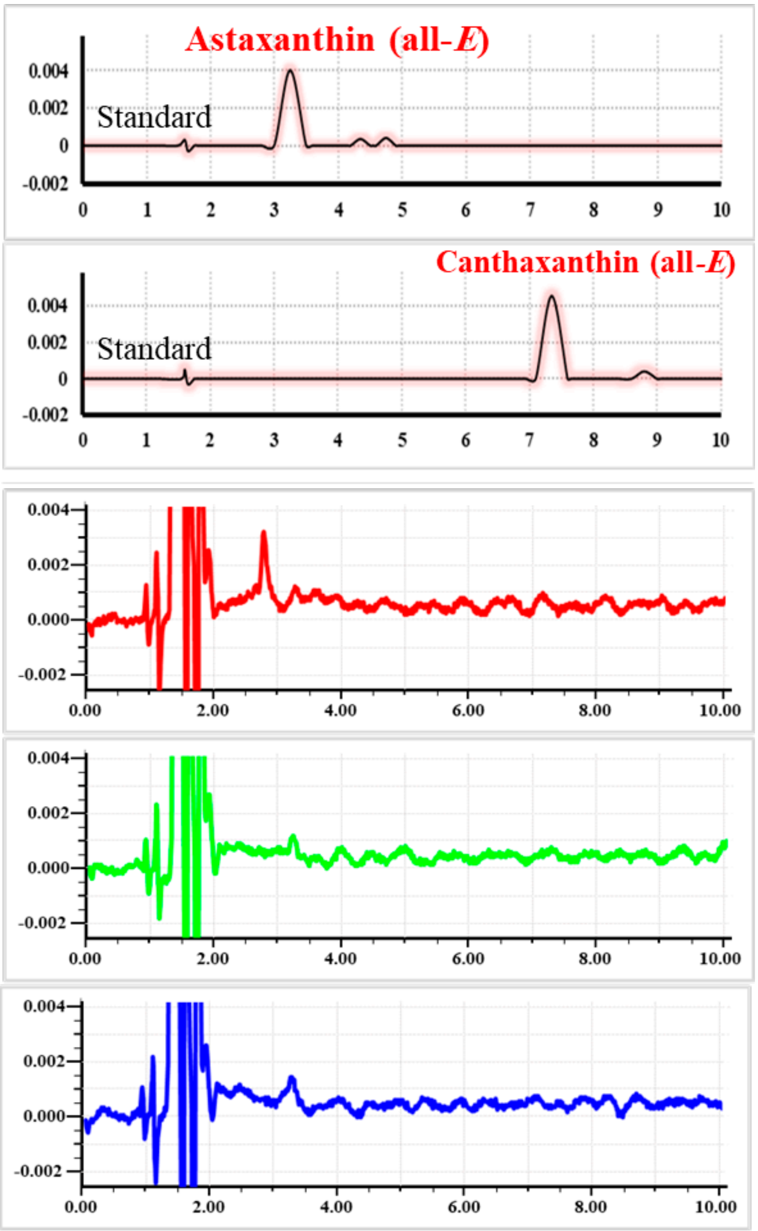

6- pPLXG+pWZg1R

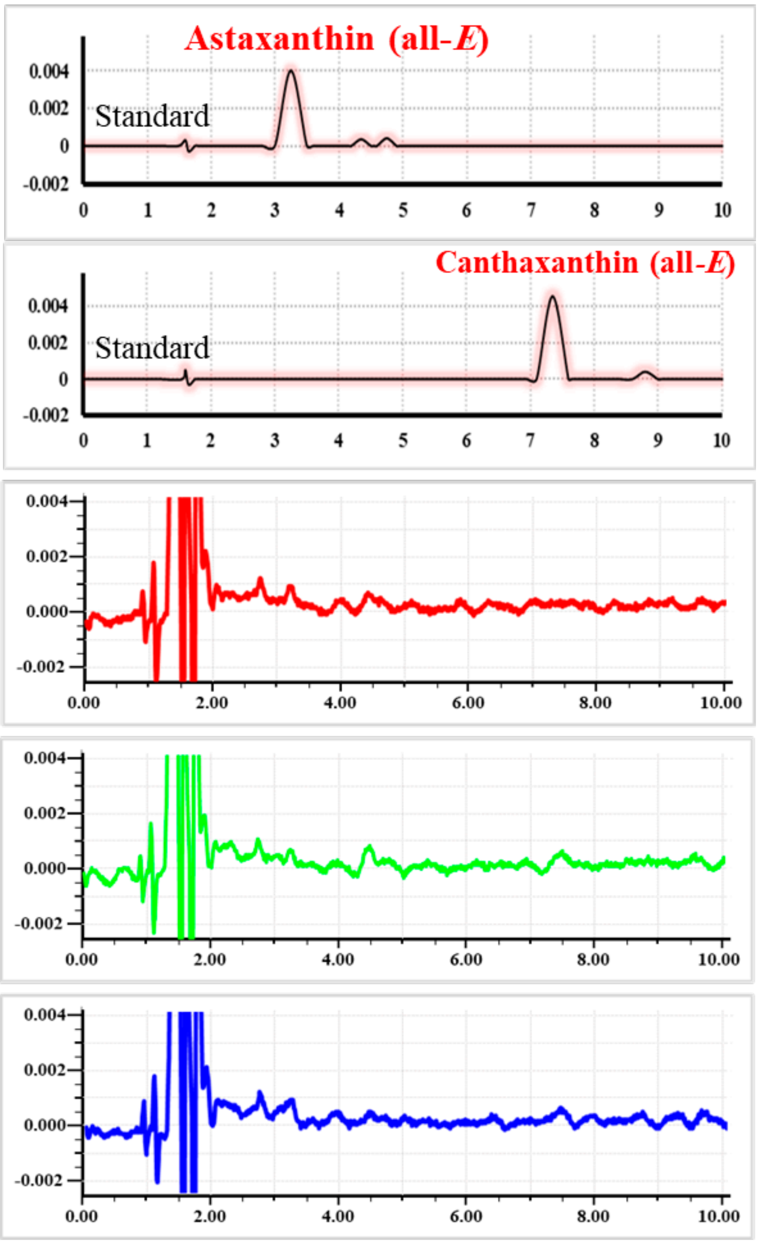

7- pAPLG+pWZg1R

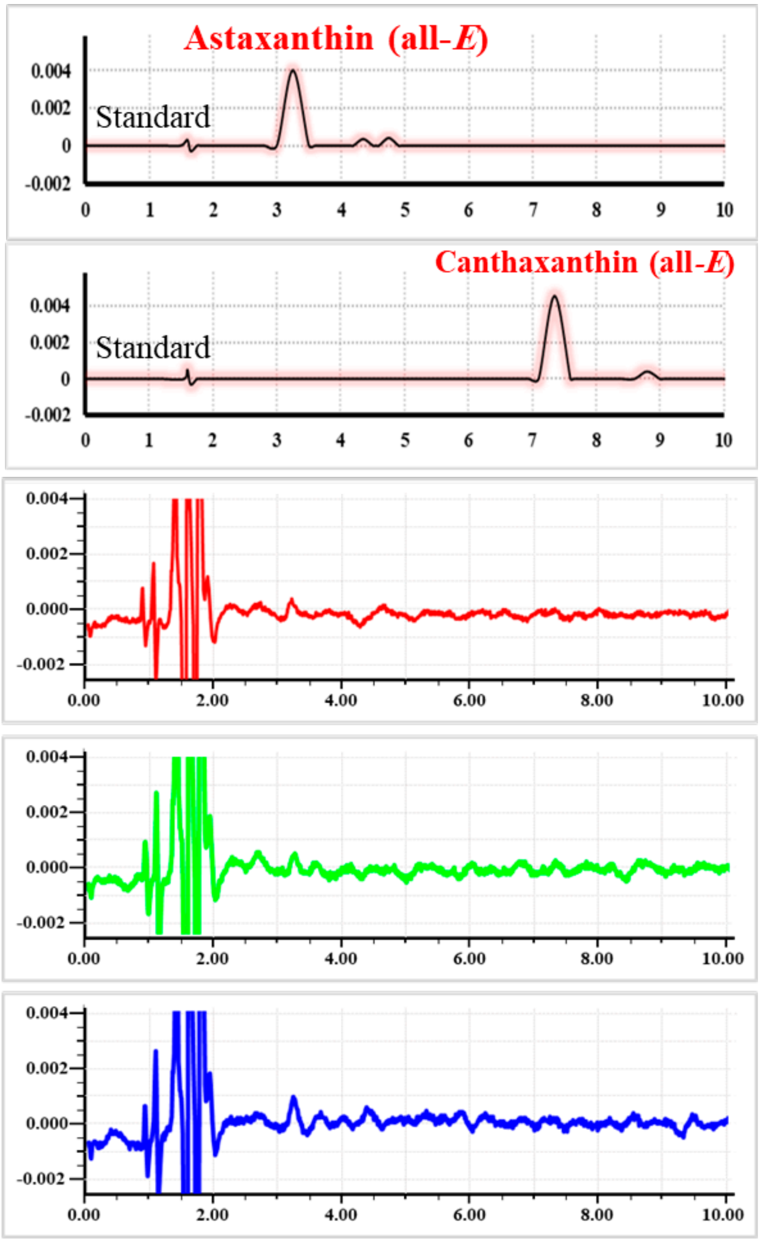

8- pPYLG+pWZg1R

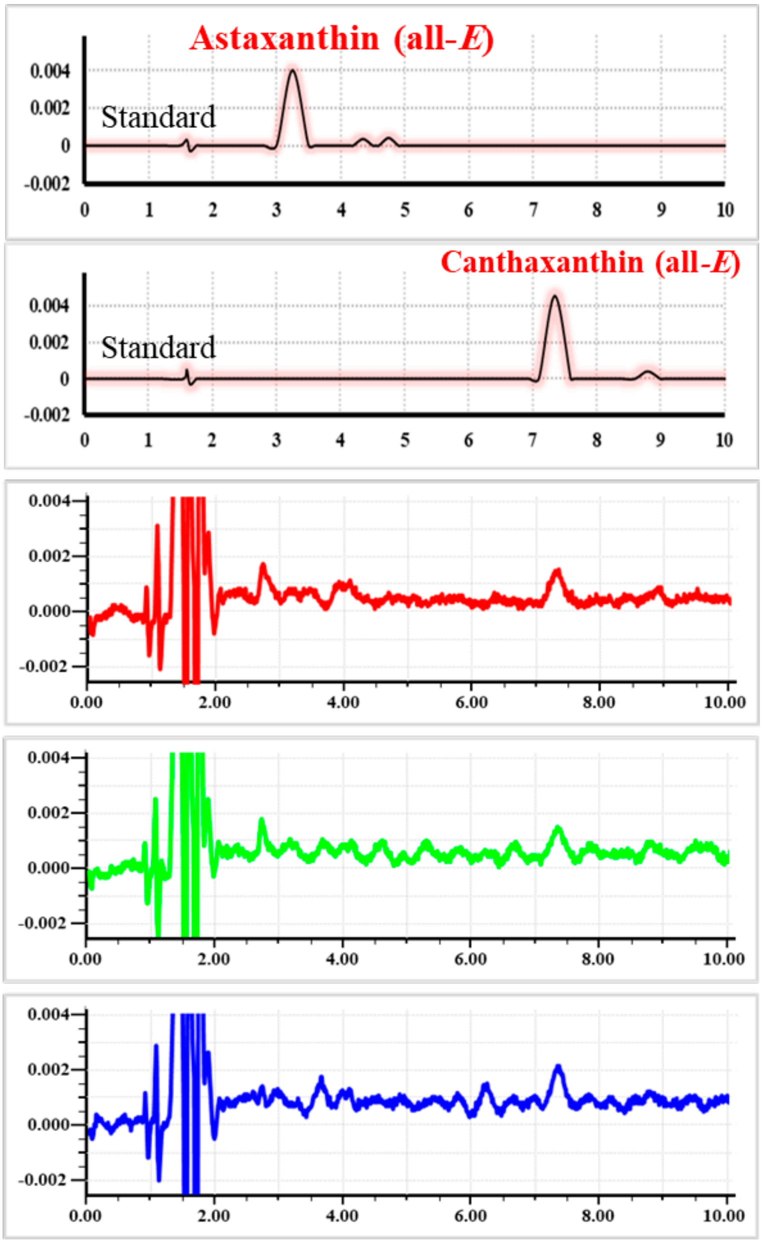

9- pPLXG+pWZg1R+GGPP

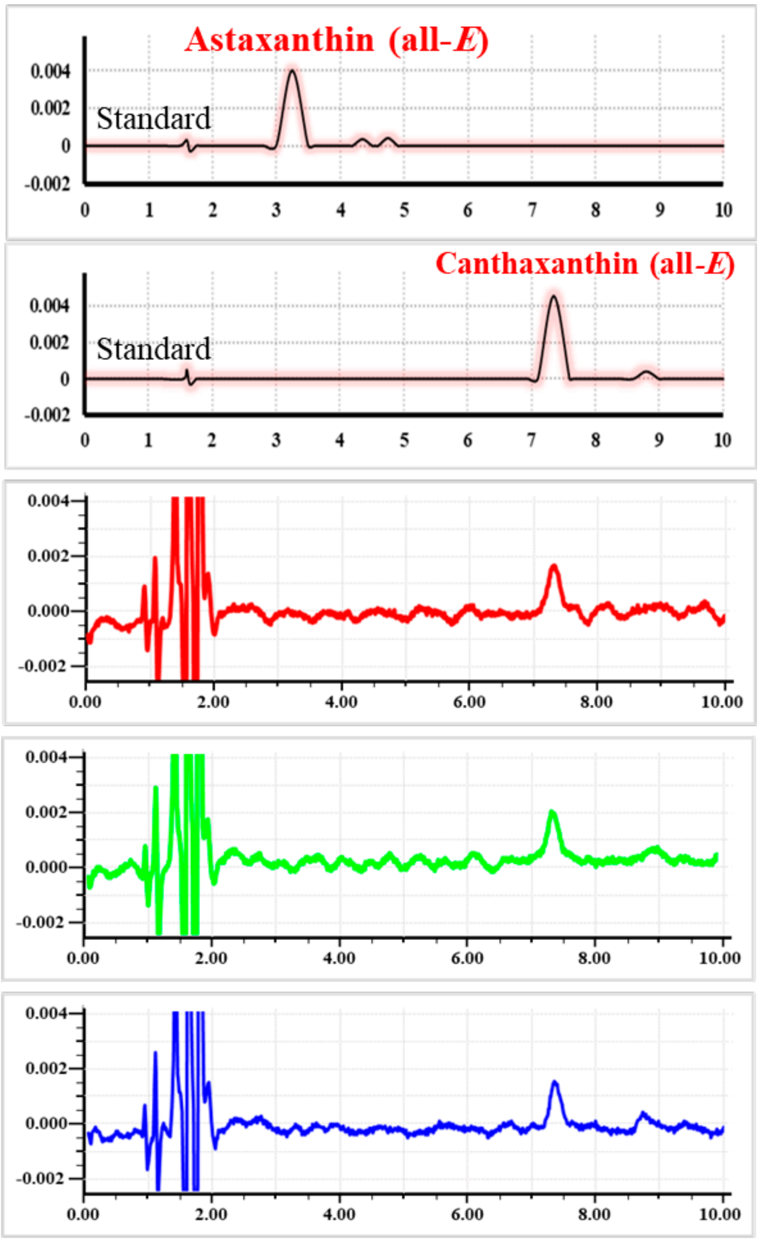

10- pAPLG+pWZg1R+GGPP

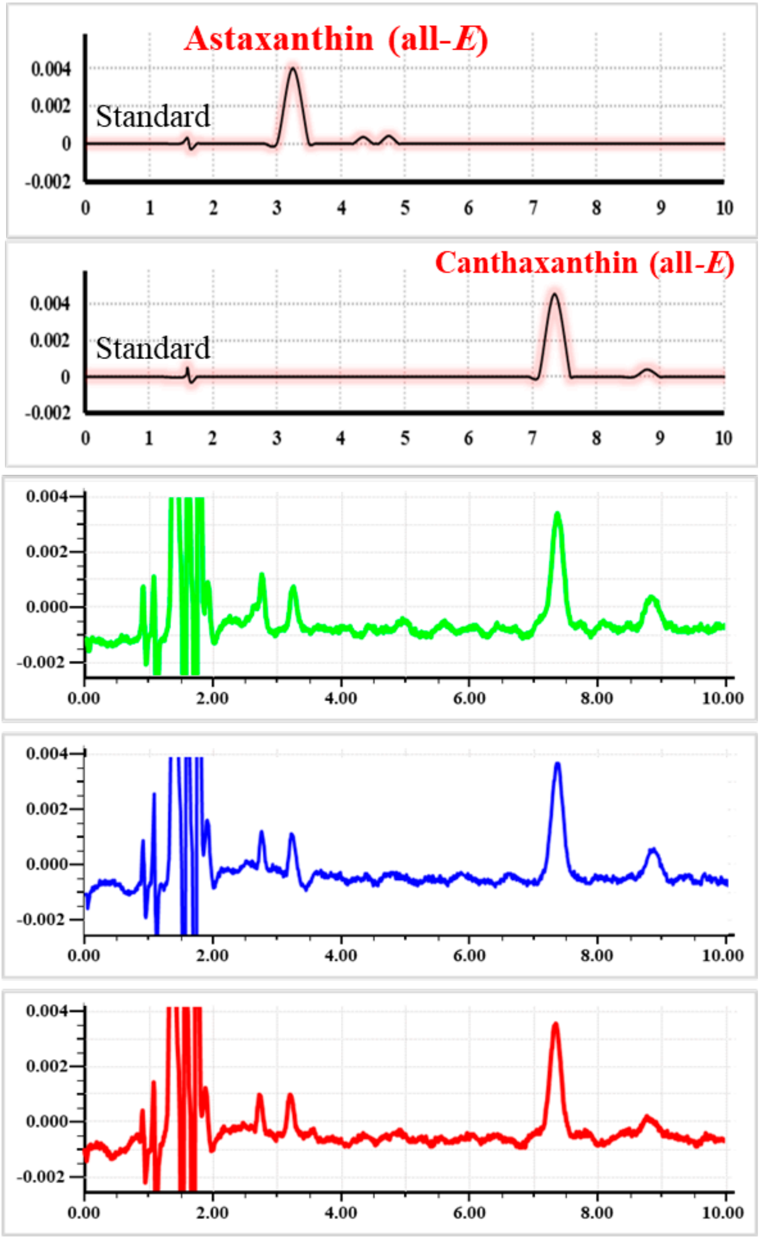

11- pPYLG+pWZg1R+GGPP

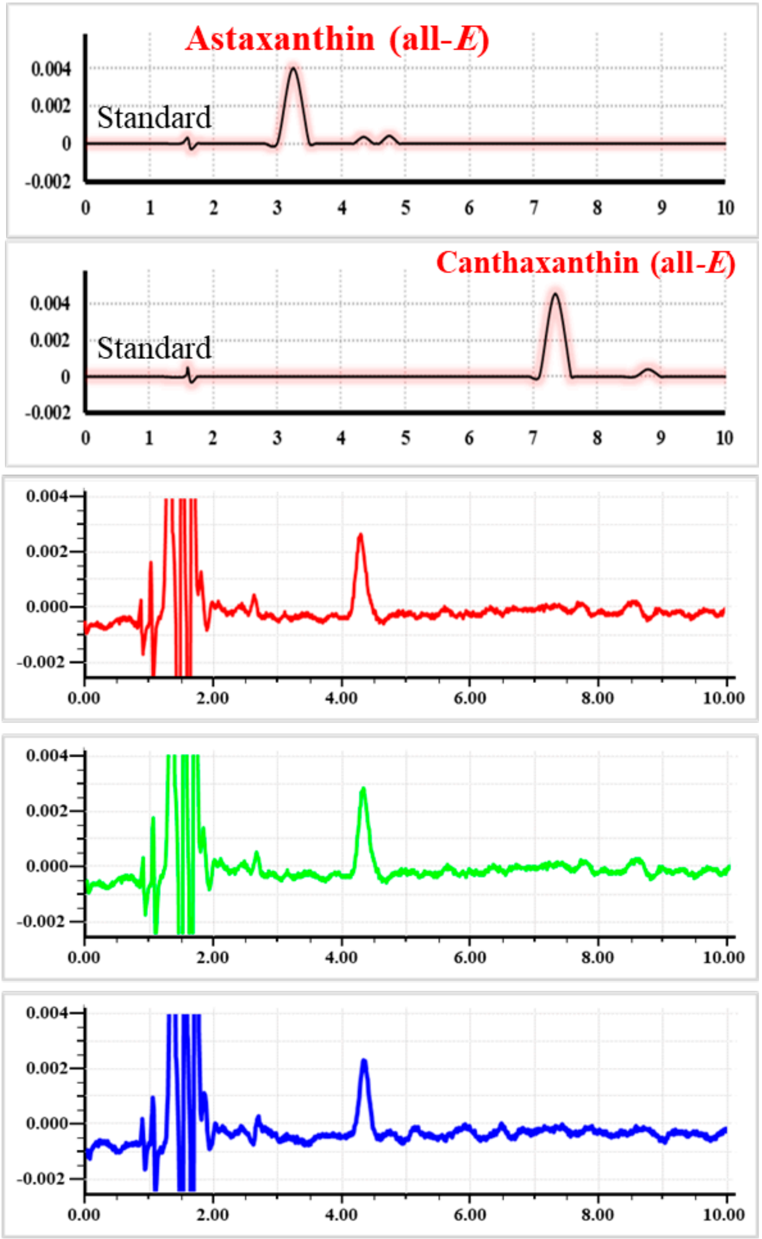

12- pPLXG+pWZg1R+ZR+GGPP

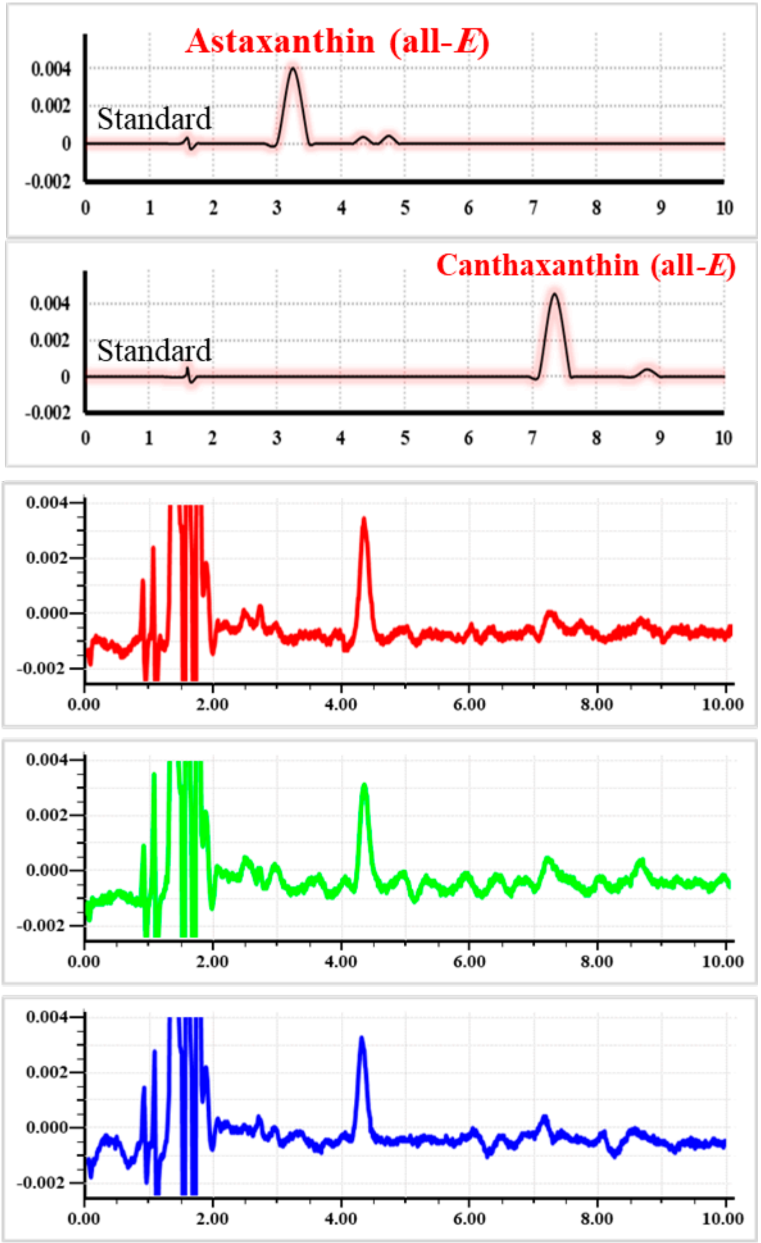

13- pAPLG+pWZg1R+ZR+GGPP

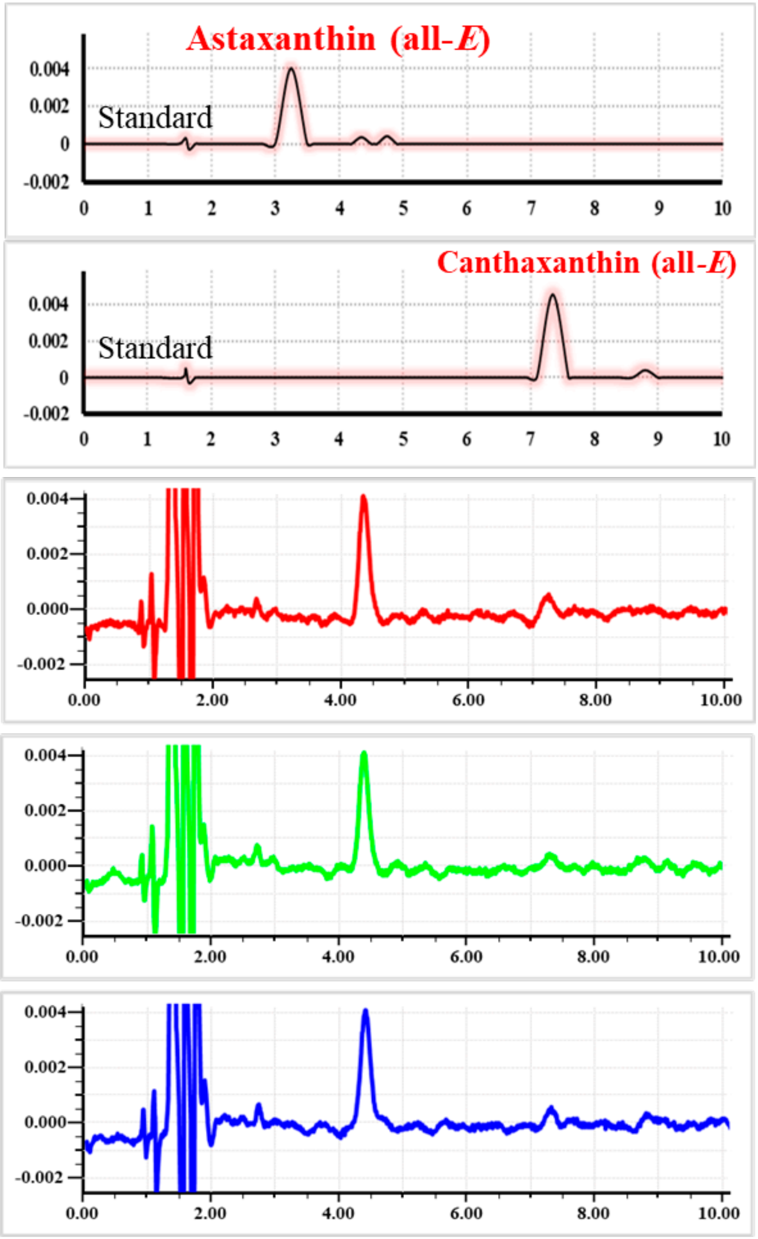

14- pPYLG+pWZg1R+ZR+GGPP
