# Supplementary material for: Production of Astaxanthin by Animal Cells via Introduction of an Entire Astaxanthin Biosynthetic Pathway
Source: Bioengineering (Basel). 2023 Sep 11;10(9):1073. doi: 10.3390/bioengineering10091073 (PMC10525450; doi:10.3390/bioengineering10091073)
Supplement: Supplementary file 1 [file bioengineering-10-01073-s001.zip › Supplementary-Table S1. Details of primers and plasmids.pdf]

Table S1. Details of primers and plasmids used in this study

Plasmid Name	Backbone	References	Primer	Primer sequence	Plasmid map
pWZG-2A	pTol2-CMV-SV40 poly(A); CMV: EGFP-SV40 poly(A)	(Pang et al., 2014)	*F..crtW*	CCATCGATATGACCGCTGCTGTGGCTGAG	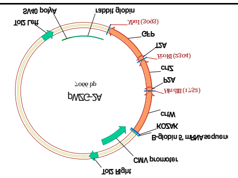
			R.crtW	CCCAAGCTTGCTCTCTCCTCTCCACAGTCT	
			F.crtZ	CCCAAGCTTGGATCCGGAGCCACGAACTTC TCTCTGTTAAAGCAAGCAGGAGACGTGGAA GAAAACCCCGGTCCTGCCTGGCTGACTTGGA TCGC	
			R.crtZ	CGGAATTCAGCTCCGCTGCTAGAAGATCCT	
pWZg1R			*R.Gtt.2*	CCATCGATCTGGATCTCCAGTGGCTTGT	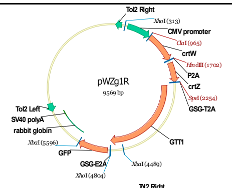
			F.Gtt.2	CGGAATTCGGATCCGGAGAGGGCAGGGGAA GTCTTCTAACATGCGGGGACGTGGAGGAAAA TCCCGGCCCCGGAGTGGCTACAAACGCTACA	
pWZg2R			*F.Gtt.1*	GGACTAGTGGATCCGGAGAGGGCAGGGGAA GTCTTCTAACATGCGGGGACGTGGAGGAAAA TCCCGGCCCCAGAATCATGCCCTGGCCTTT	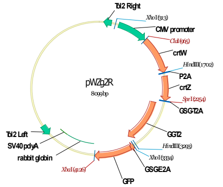
			R.Gtt.1	CCGCTCGAGGATCACGATTCCAGAGCTTGT	
pbK3HZ-2A			*F. bkt3*	CCATCGATATGCACGTGGCTAGCGCTCT	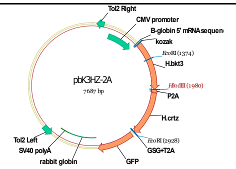
			R. bkt3	CCCAAGCTTAGCCAGAGCAGGCACCAGT	
			F.HcrtZ	CCCAAGCTTGGATCCGGAGCCACGAACTT CTCTCTGTTAAAGCAAGCAGGAGACGTGG AAGAAAACCCCGGTCCTCTCAGCAAGCTG CAGAGCAT	
			R.H.crtZ	CGGAATTCTCTTTTGCTCCAGTCCAGTT	
pC1HZG-2A			*F.Cbkt1*	CCATCGATATGGGACCTGGAATCCAGCCGA	
			R.Cbkt1	CCCAAGCTTAGCCATAACCCCCACAGGA	
			F.GFP	CGGAATTCGGATCCGGAGAGGGCAGGGGA	

				AGTCTTCTAACATGCGGGGACGTGGAGGA AAATCCCGGCCCCGTGAGCAAGGGCGAG GAGCT	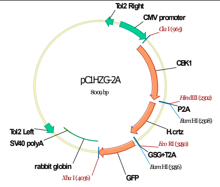
			R.GFP	GCTCTAGATTACTTGTACAGCTCGTCCAT	
			F.mcherry	CGGAATTCGGATCCGGAGAGGGCAGGGG AAGTCTTCTAACATGCGGGGACGTGGAG GAAAATCCCGGCCCCGTGAGCAAGGGCG AGGAGGAT	
			R.mcherry	GCTCTAGATTACTTGTACAGCTCGTCCAT	
pPYLG			*F.PSY1*	CCATCGATATGGCAATCATCCTGGTGAGA	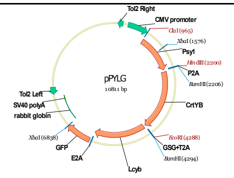
			R.PSY1	CCCAAGCTTTGTCTGTCCGTTTCTCAGGCT	
			F.CrtYB	CCCAAGCTTGGATCCGGAGCCACGAACTT CTCTCTGTTAAAGCAAGCAGGAGACGTGG AAGAAAACCCCGGTCCTACAGCCCTGGCC TACTACCA	
			R.CrtYB	CGGAATTCCTGTCCCTCCCATCCACTCA	
			F.Lcyb1	CCCAAGCTTGGATCCGGAGCCACGAACTT CTCTCTGTTAAAGCAAGCAGGAGACGTGG AAGAAAACCCCGGTCCTCTGAGCCCTCTG CAGAGACT	
			R.Lcyb1	CGGAATTCCTTGATCATAGCGGGTGGCA	
pPLXG			*F.xd.crtI*	CGGAATTCGGATCCGGAGAGGGCAGGGGA AGTCTTCTAACATGCGGGGACGTGGAGGAA AATCCCGGCCCCGGAAAGGAGCAGGACCA GGA	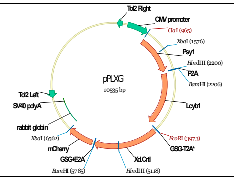
			R.xd.CrtI	CGGGATCCGAAAGCCAGCACTCCCACG	
pAPLG			*F.Pa.crtI*	CCATCGATATGAAGCCCACAACCGTGAT	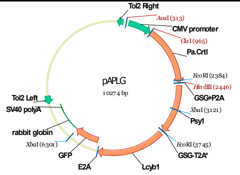
			R.Pa.crtI	CCCAAGCTTGATCAGGTCTTCCAGCATCA	
			F.Pa.psy1	CCCAAGCTTGGATCCGGAGCCACGAACTTC TCTCTGTTAAAGCAAGCAGGAGACGTGGAA GAAAACCCCGGTCCTGCAATCATCCTGGTG AGAG	
			R.Pa.psy1	CGGAATTCTGTCTGTCCGTTTCTCAGG	

			F.Pa.Lcyb1	CGGAATTCGGATCCGGAGAGGGCAGGGGAA GTCTTCTAACATGCGGGGACGTGGAGGAAAA TCCCGGCCCCCTGAGCCCTCTGCAGAGACT	
			R.Pa.Lcyb1	CGGGATCCCTTGATCATAGCGGGTGGCA	
			F.GFP	CGGGATCCCGGATCCGGACAGTGTACTA ATTATGCTCTCTTGAAATTGGCTGGAGAT GTTGAGAGCAACCCAGGTCCCGTGAGCA AGGGCGAGGAGCT	
			R.GFP	GCTCTAGATTACTTGTACAGCTCGTCCAT	
