# Supplementary material for: Production of Astaxanthin by Animal Cells via Introduction of an Entire Astaxanthin Biosynthetic Pathway
Source: Bioengineering (Basel). 2023 Sep 11;10(9):1073. doi: 10.3390/bioengineering10091073 (PMC10525450; doi:10.3390/bioengineering10091073)
Supplement: Supplementary file 1 [file bioengineering-10-01073-s001.zip › Supplementary-Table S2. Compositions and contents of total astaxanthin in HEK293T cells - modified.pdf]

Table S2. Compositions and contents of total astaxanthin in HEK293T cells. Data (µg/g DW, gram of dry weight).

Samples	Ast-E	Ast-Z	Total-Ast	MIN-TOTLA-AST	SD	Compared to	One way ANOVA, *P-values*
	ASTX µg/g DW	µg/g DW	µg/g DW	µg/g DW			
**pbK3HZ-2A+beta-carotene**	ND	9.4	9.4	3.1	5.4	pWZG-2A+beta-carotene	
	ND	ND	ND				
	ND	ND	ND				
**pC1HZG-2A+beta-carotene**	19.3	ND	19.3	17.8	1.5	pbK3HZ-2A+beta-carotene	**
	16.3	ND	16.3				
	17.8	ND	17.8				
**pWZG-2A+beta-carotene**	32.9	ND	32.9	31.6	1.3	pbK3HZ-2A+beta-carotene	**
	30.3	ND	30.3				
	31.6	ND	31.6				
**pWZg2R-2A+beta-carotene**	31.3	ND	31.3	32.5	1.3	pWZG-2A+beta-carotene	
	33.8	ND	33.8				
	32.5	ND	32.5				
**pWZg1R-2A+beta-carotene**	25.2	ND	25.2	34.1	8.5	pWZG-2A+beta-carotene	
	35.0	ND	35.0				
	12.5	29.6	42.1				
**pPLXG+pWZg1R**	17.9	ND	17.9	21.7	5.1	pPYLG+pWZg1R	
	19.7	ND	19.7				
	27.6	ND	27.6				
**pAPLG+pWZg1R**	12.2	21.1	33.3	31.4	2.0	pPLXG+pWZg1R	*
	ND	31.6	31.6				
	ND	29.3	29.3				
**pPYLG+pWZg1R**	21.4	19. 2	40.6	41.9	1.3	pPLXG+pWZg1R	***
	22.8	20.4	43.2				
	ND	41.8	41.8				
**pPLXG+pWZg1R+GGPP**	19.7	ND	19.7	19.5	1.1	pPLXG+pWZg1R	***
	18.3	ND	18.3				
	20.5	ND	20.5				
**pAPLG+pWZg1R+GGPP**	21.7	11.6	33.2	43.2	8.7	pAPLG+pWZg1R	***
	40.0	7.5	47.4				
	41.5	7.5	49.0				
**pPYLG+pWZg1R+GGPP**	38.7	20.4	59.1	54.1	5.0	pPYLG+pWZg1R	*
	49.2	ND	49.2				
	44.8	9.2	54.5				
**pPLXG+pWZg1R+ZR+GGPP**	ND	152.4	152.4	146.4	7.8	pPLXG+pWZg1R	***
	ND	137.6	137.6				
	ND	149.2	149.2				
**pAPLG+pWZg1R+ZR+GGPP**	ND	134.7	134.7	136.7	2.1	pAPLG+pWZg1R	***
	ND	138.8	138.8				

	ND	136.7	136.7				
**pPYLG+pWZg1R+ZR+GGPP**	ND	239.3	239.3	232.8	6.0	pPYLG+pWZg1R	***
	ND	227.6	227.6				
	ND	231.5	231.5				

P < 0.05, P < 0.01 and P < 0.001 are tagged as *, **, and ***, respectively. ND, not detectable. Astaxanthin is the sum of *E (trans)* and *Z (cis)* isomers.
