# Supplementary material for: Production of Astaxanthin by Animal Cells via Introduction of an Entire Astaxanthin Biosynthetic Pathway
Source: Bioengineering (Basel). 2023 Sep 11;10(9):1073. doi: 10.3390/bioengineering10091073 (PMC10525450; doi:10.3390/bioengineering10091073)
Supplement: Supplementary file 1 [file bioengineering-10-01073-s001.zip › Supplementary-Table S3. Compositions and contents of total canthaxanthin in HEK293T cells - modified.pdf]

Table S3. Compositions and contents of total canthaxanthin in HEK293T cells. Data (μg/g DW, gram of dry weight).

Samples	CAN-E	CAN-Z	TOTLA-CAN	MIN-Total-CAN	SD	Compared to	One way ANOVA, *P-values*
	μg/g DW	μg/g DW	μg/g DW	μg/g DW			
**pbK3HZ-2A+beta-carotene**	39.6	ND	39.6	37.1	2.4	pWZG-2A+beta-carotene	
	34.8	ND	34.8				
	36.9	ND	36.9				
**pC1HZG-2A+beta-carotene**	37.2	ND	37.2	37.8	8.0	pbK3HZ-2A+beta-carotene	*
	32.0	14.2	46.1				
	30.1	ND	30.1				
**pWZG-2A+beta-carotene**	42.3	ND	42.3	42.6	1.5	pbK3HZ-2A+beta-carotene	***
	41.3	ND	41.3				
	44.2	ND	44.2				
**pWZg2R-2A+beta-carotene**	25.1	32.6	57.6	57.9	0.6	pWZG-2A+beta-carotene	
	25.6	32.9	58.5				
	24.2	33.2	57.5				
**pWZg1R-2A+beta-carotene**	83.6	31.6	115.2	106.1	12.0	pWZG-2A+beta-carotene	***
	81.7	29.0	110.6				
	92.5	ND	92.5				
**pPLXG+pWZg1R**	20.2	ND	20.2	21.8	1.3	pPYLG+pWZg1R	
	ND	22.7	22.7				
	22.3	ND	22.3				
**pAPLG+pWZg1R**	ND	17.5	17.5	20.3	2.5	pPLXG+pWZg1R	
	11.8	10.3	22.1				
	21.5	ND	21.5				
**pPYLG+pWZg1R**	19.0	ND	19.0	21.4	2.1	pPLXG+pWZg1R	
	22.1	ND	22.1				
	23.0	ND	23.0				
**pPLXG+pWZg1R+GGPP**	21.3	37.4	58.8	55.4	3.5	pPLXG+pWZg1R	
	37.2	14.6	51.8				
	15.0	40.5	55.5				
**pAPLG+pWZg1R+GGPP**	19.5	67.0	86.5	81.2	5.1	pAPLG+pWZg1R	*
	38.9	37.4	76.3				
	44.9	35.8	80.8				
**pPYLG+pWZg1R+GGPP**	133.0	20.6	153.5	153.1	0.5	pPYLG+pWZg1R	***
	124.6	28.7	153.3				
	114.0	38.5	152.5				
**pPLXG+pWZg1R+ZR+GGPP**	8.7	ND	8.7	8.2	1.2	pPLXG+pWZg1R	***
	9.1	ND	9.1				
	6.8	ND	6.8				
**pAPLG+pWZg1R+ZR+GGPP**	56.9	ND	56.9	59.2	8.0	pAPLG+pWZg1R	**
	52.6	ND	52.6				

	68.1	ND	68.1				
**pPYLG+pWZg1R+ZR+GGPP**	23.7	4.0	27.7	29.3	2.0	pPYLG+pWZg1R	**
	20.1	11.4	31.5				
	28.7	ND	28.7				

$P < 0.05$, $P < 0.01$ and $P < 0.001$ are tagged as *, **, and ***, respectively. ND, not detectable. The canthaxanthin is the sum of E *(trans)* and *Z (*cis) isomers.
