# Supplementary material for: Production of Astaxanthin by Animal Cells via Introduction of an Entire Astaxanthin Biosynthetic Pathway
Source: Bioengineering (Basel). 2023 Sep 11;10(9):1073. doi: 10.3390/bioengineering10091073 (PMC10525450; doi:10.3390/bioengineering10091073)
Supplement: Supplementary file 1 [file bioengineering-10-01073-s001.zip › Supplementary-Table S4. Compositions and contents of total-ketocarotenoids in HEK293T cells - modified.pdf]

**Table S4.** Compositions and contents of total-ketocarotenoids in HEK293T cells. Data (μg/g DW, gram of dry weight).

| Samples | TOTAL-KETOCAROTEN | MIN-total-keto | SD | Compared to | One way ANOVA, *P-values* |
|---|---|---|---|---|---|
| | μg/g DW | μg/g DW | | | |
| **pbK3HZ-2A+beta-carotene** | 49.0 | 40.2 | 7.6 | pWZG-2A+beta-carotene | |
| | 34.8 | | | | |
| | 36.9 | | | | |
| **pC1HZG-2A+beta-carotene** | 56.6 | 55.6 | 7.3 | pbK3HZ-2A+beta-carotene | |
| | 62.4 | | | | |
| | 47.9 | | | | |
| **pWZG-2A+beta-carotene** | 75.1 | 74.2 | 2.2 | pbK3HZ-2A+beta-carotene | |
| | 71.7 | | | | |
| | 75.8 | | | | |
| **pWZg2R-2A+beta-carotene** | 88.9 | 90.4 | 1.8 | pWZG-2A+beta-carotene | ** |
| | 92.4 | | | | |
| | 90.0 | | | | |
| **pWZg1R-2A+beta-carotene** | 140.4 | 140.2 | 5.5 | pWZG-2A+beta-carotene | *** |
| | 145.6 | | | | |
| | 134.6 | | | | |
| **pPLXG+pWZg1R** | 38.2 | 43.5 | 5.9 | pPYLG+pWZg1R | |
| | 42.4 | | | | |
| | 49.9 | | | | |
| **pAPLG+pWZg1R** | 50.8 | 51.7 | 1.7 | pPLXG+pWZg1R | |
| | 53.7 | | | | |
| | 50.8 | | | | |
| **pPYLG+pWZg1R** | 59.6 | 63.2 | 3.1 | pPLXG+pWZg1R | ** |
| | 65.3 | | | | |
| | 64.8 | | | | |
| **pPLXG+pWZg1R+GGPP** | 78.4 | 74.9 | 4.2 | pPLXG+pWZg1R | ** |
| | 70.2 | | | | |
| | 76.0 | | | | |
| **pAPLG+pWZg1R+GGPP** | 119.7 | 124.4 | 5.0 | pAPLG+pWZg1R | *** |
| | 123.7 | | | | |
| | 129.8 | | | | |
| **pPYLG+pWZg1R+GGPP** | 212.6 | 207.2 | 5.1 | pPYLG+pWZg1R | *** |
| | 202.4 | | | | |
| | 206.5 | | | | |
| **pPLXG+pWZg1R+ZR+GGPP** | 161.1 | 154.6 | 7.3 | pPLXG+pWZg1R | *** |
| | 146.7 | | | | |
| | 156.0 | | | | |
| **pAPLG+pWZg1R+ZR+GGPP** | 191.6 | 196.0 | 7.7 | pAPLG+pWZg1R | *** |
| | 191.5 | | | | |
| | 204.8 | | | | |
| **pPYLG+pWZg1R+ZR+GGPP** | 267.1 | 262.1 | 4.3 | pPYLG+pWZg1R | *** |

| | | | | | |
|---|---|---|---|---|---|
| | 259.0 | | | | |
| | 260.2 | | | | |

$P < 0.05$, $P < 0.01$ and $P < 0.001$ are tagged as *, **, and ***, respectively. ND, not detectable. The total-ketocarotenoids is the sum of E *(trans)* and *Z (*cis) isomers.
